# Low-dose “metronomic chemotherapy” with oral cyclophosphamide and methotrexate in metastatic breast cancer: a case report of extraordinarily prolonged clinical benefit

**DOI:** 10.3332/ecancer.2012.275

**Published:** 2012-10-18

**Authors:** G Masci, A Losurdo, C Gandini, I Garassino, L di Tommaso, R Torrisi, M Zuradelli, A Santoro

**Affiliations:** 1 Humanitas Cancer Center, Istituto Clinico Humanitas, Istituto Di Ricovero e Cura a Carattere Scientifico, Via Manzoni 56, 20089 Rozzano, Milan, Italy

## Abstract

We report the case of a 34-year-old woman affected by breast cancer that had metastasized to the bone. She had been treated with oral cyclophosphamide and methotrexate (metronomic chemotherapy) and achieved 3.5 years of clinical remission. To our knowledge, this is the first description of such a prolonged response to therapy. This case report adds weight to known data on metronomic treatment and supports further investigation of this therapy.

## Introduction

**Figure 1:**
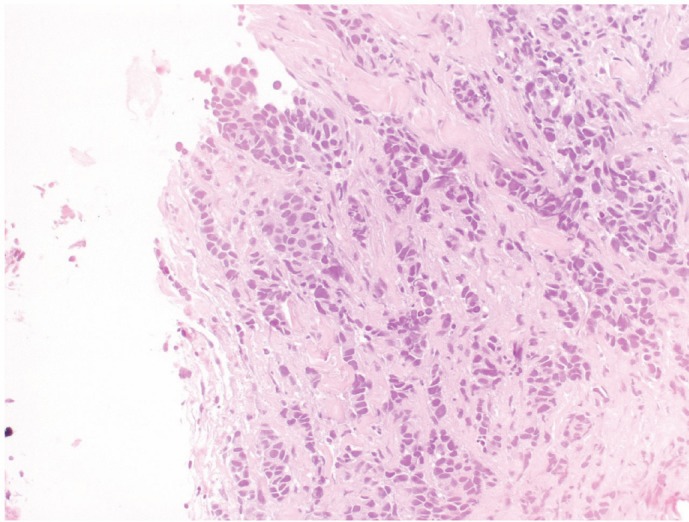
Neoplastic cells growing in poorly defined glandular structures were seen at the periphery of the biopsy (H&E).

**Figure 2:**
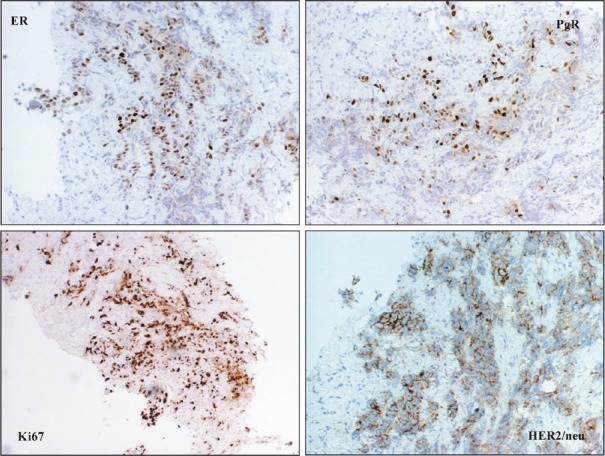
Neoplastic cells were positive for ER, PgR and HER2/neu and are characterized by a high proliferative fraction.

A 34-year-old woman was diagnosed with left breast cancer and metastatic ipsilateral axilla lymph nodes in August 2004. A tru-cut biopsy of the breast lump confirmed that it was an invasive carcinoma (Figure 1), the tissue was positive for hormonal estrogen and progesterone receptors (50%), HER-2 expression was complete and intense in 70% of neoplastic cells (3+), and Ki-67 expression was positive in 20% off cells (Figure 2). A bone scan indicated secondary bone lesions (sternum, left humeral head, T10, L4, and the skull). The serum Ca 15.3 level was 123 UI/ml (normal range 1–25 UI/ml). Six cycles of adriamycin and cyclophsphamide were administered.

At the end of the adriamycin regimen, tamoxifen (20 mg daily) was started and the patient exhibited stable disease. Disease response was evaluated through ultrasound of the breast and axilla and bone scan of the metastases.

In May 2007, a bone scan indicated that a new lesion had formed on the first rib and the tumour marker (Ca15-3) levels had increased. Thus, the hormonal therapy was switched from tamoxifen to anastrozole.

**Figure 3:**
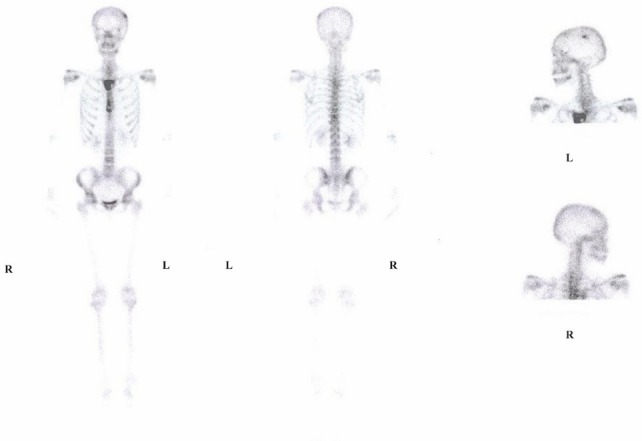
Bone scan before oral Cyclophosphamide and Methotrexate chemotherapy.

In May 2008, the breast site had progressed, and a new lesion was detected on the skull (Figure 3). The patient was offered treatment with taxane and trastuzumab but refused because she was unwilling to undergo further IV treatments.

We therefore administered metronomic oral cyclophosphamide and methotrexate (CM) according to the schedule described by Colleoni [1]. Cyclophosphamide was administered at 50 mg/day and methotrexate at 2.5 mg, twice a day, on days 1 and 4 each week.

**Figure 4:**
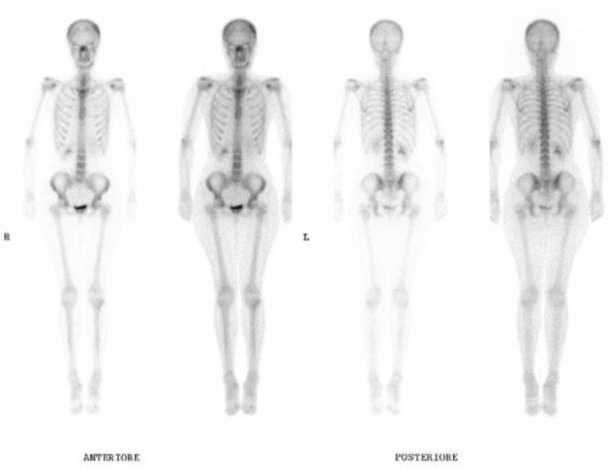
Bone scan after 42 months of oral Cyclophosphamide and Methotrexate chemotherapy.

After six months of treatment, the tumour marker (Ca15-3) had progressively decreased to normal levels (from 86 to 16 UI/ml). In addition, the mammary lesion had shrunk to the size of a crease in the skin, and no evidence of disease was detected at the last ultrasound. The last bone scan showed a pathologic capitation only on the skull and sternum (Figure 4).

As of November 2011, the patient has completed 42 months of CM therapy, with continuous progression-free survival. There was no reported side effect related to the CM schedule. The patient is alive to date with stable disease (7-year overall survival).

## Discussion

Metastatic breast cancer is a heterogeneous disease, and decisions regarding its treatment must take into considerations not only the clinical and biological parameters of the case but also the patient’s preferences.

Metronomic chemotherapy comprised chronic administration of a chemotherapeutic agent at relatively low, non-toxic doses, with no prolonged, drug-free breaks. This is a potentially novel approach for controlling advanced cancer [2]. In some studies, metronomic chemotherapy was shown to be effective alone [1, 3–4], or in combination with letrozole [5], trastuzumab [6], or bevacizumab [7].

Metronomic chemotherapy is thought to exert anticancer activity, primarily by inhibiting tumour angiogenesis [8]. In Colleoni’s study [1], a drop in vascular endothelium growth factor (VEGF) concentration was observed in the serum of patients undergoing treatment with CM schedule, although the authors themselves caution against taking these findings at face value, as other factors may concur to the reported VEGF decrease.

An important contribution to the understanding of the angiogenic activity associated with metronomic chemotherapy was from a study by Browder *et al * [9]. In that study, the maximum tolerated dose (MTD) of cyclophosphamide was administered at the tumour-bearing mice. The result was significant endothelial-cell apoptotic death in tumour-associated microvasculature. However, this potentially desirable drug effect did not seem to translate into a significant therapeutic benefit, because the damage to tumour vasculature was repaired during the 2-3-week rest periods between successive cycles of MTD-based therapy. This suggested that chronic cyclophosphamide therapy might effectively prevent repair of tumour endothelium.

To our knowledge, the current study is the first report of prolonged remission in a patient with metastatic breast cancer treated with a CM schedule. Although this patient’s disease was indolent with no visceral metastases, it was a notoriously aggressive, HER-2 over-expressing tumour. Therefore, the response to CM, which has lasted over three years, has been quite impressive. This led to the hypothesis that using a metronomic regimen, in limited metastatic disease, may prevent resistance to chemotherapy, and thus promote a long-term response.

We are currently considering the important question of whether we should change the treatment. Generally, in clinical practice, a treatment is discontinued when it is no longer effective or when limiting toxicity occurs. In this case, neither condition has been observed, but there is a risk of leukemia associated with prolonged use of alkylating agents. In study by Curtis [10], the risk among patients treated with over 20,000 mg of cyclophosphamide was 5.7 times that of women not treated with alkylating agents. Furthermore, the duration of therapy was also strongly associated with the risk of leukemia. In our case, the dose of cyclophosphamide was fairly high (54,000 mg): therefore, the prolonged use of the CM regimen will require careful consideration.

Based on the results of this case report, in addition to other published data, we recommend that metronomic chemotherapy should be further evaluated. In particular, future studies should investigate its potential value as a first-line treatment.
